# ABCG2 and SLC1A5 functionally interact to rewire metabolism and confer a survival advantage to cancer cells under oxidative stress

**DOI:** 10.1016/j.jbc.2024.107299

**Published:** 2024-04-18

**Authors:** Jia Shi, Kirk Pabon, Rui Ding, Kathleen W. Scotto

**Affiliations:** 1 Department of Pharmacology, Robert Wood Johnson Medical School, Rutgers Biomedical Health Sciences, Rutgers, The State University of New Jersey, Piscataway, New Jersey, USA; 2 Clinical Pharmacology, Translational Medicine, Servier Pharmaceuticals LLC, Boston, Massachusetts, USA

**Keywords:** ABCG2, SLC1A5, cancer metabolism, oxidative stress, survival advantage, breast cancer, autophagy, glutamine metabolism

## Abstract

ABCG2, a member of the ABC transporter superfamily, is overexpressed in many human tumors and has long been studied for its ability to export a variety of chemotherapeutic agents, thereby conferring a multidrug resistance (MDR) phenotype. However, several studies have shown that ABCG2 can also confer an MDR-*independent* survival advantage to tumor cells exposed to stress. While investigating the mechanism by which ABCG2 enhances survival in stressful milieus, we have identified a physical and functional interaction between ABCG2 and SLC1A5, a member of the solute transporter superfamily and the primary transporter of glutamine in cancer cells. This interaction was accompanied by increased glutamine uptake, increased glutaminolysis, and rewired cellular metabolism, as evidenced by an increase in key metabolic enzymes and alteration of glutamine-dependent metabolic pathways. Specifically, we observed an increase in glutamine metabolites shuttled to the TCA cycle, and an increase in the synthesis of glutathione, accompanied by a decrease in basal levels of reactive oxygen species and a marked increase in cell survival in the face of oxidative stress. Notably, the knockdown of SLC1A5 or depletion of exogenous glutamine diminished ABCG2-enhanced autophagy flux, further implicating this solute transporter in ABCG2-mediated cell survival. This is, to our knowledge, the first report of a functionally significant physical interaction between members of the two major transporter superfamilies. Moreover, these observations may underlie the protective role of ABCG2 in cancer cells under duress and suggest a novel role for ABCG2 in the regulation of metabolism in normal and diseased states.

There are two human transporter superfamilies, the ABC (ATP-Binding Cassette) transporter family and the SLC (Solute Carrier) transporter family. The 48 ABC transporters include proteins that hydrolyze ATP to efflux various metabolites, signaling molecules, and xenobiotics (including chemotherapeutics) across cell membranes under both physiological and pathological conditions. The SLC superfamily contains over 400 transporters involved in the transfer of ions, nucleotides, sugars, and amino acids. Members of both the ABC and SLC transporter families are selectively expressed in a variety of tissues, and many are overexpressed or aberrantly expressed under pathological conditions, including cancer and neurological disease.

Our laboratory has had a long-standing interest in the regulation and function of ABC transporters, with a special focus on those that are aberrantly expressed in malignant cells and have been shown to confer a survival advantage to cells exposed to a plethora of stressors, including chemotherapeutics. Our recent studies have focused on ABCG2 (BCRP), first identified due to its overexpression in multidrug-resistant breast cancer cell lines and later found to be highly expressed in a variety of human cancers including acute myelogenous leukemia, acute lymphoblastic leukemia, breast and lung cancers (1), where its overexpression has been correlated with poor prognosis. Given the well-documented ability of ABCG2 to confer a multidrug-resistant (MDR) phenotype by virtue of its ability to efflux several classes of chemotherapeutic drugs, this correlation between ABCG2 expression and poor prognosis has most often been attributed to this drug efflux activity (2). However, that mechanism cannot explain observations made in a number of preclinical studies, where ABCG2 overexpression was associated with survival following exposure to non-substrate stressors such as nutrient deprivation and radiation (3, 4). Nor does it explain observations made in the clinic, where the expression of ABCG2 has been correlated with tumor survival and poor prognosis independent of drug treatment (5, 6, 7, 8). This has led to speculation that ABCG2 has an additional function(s) that promotes tumor cell survival under stressful conditions (9, 10, 11). Indeed, we (3) and others (12, 13) have reported that ABCG2 overexpression is accompanied by an increase in autophagy flux, thereby conferring a transient but striking increase in cell survival when faced with these environmental stressors. While this observation defines a new mechanism by which ABCG2 may impact tumor cell survival, how this is accomplished and whether other mechanisms are also in play have not yet been determined.

To further define the mechanism(s) by which ABCG2 enhances tumor cell survival, we considered the possibility that ABCG2 might accomplish this through interaction with other membrane proteins. We now report that ABCG2 interacts with SLC1A5, a member of the human solute transporter superfamily and the major glutamine transporter in tumor cells. Like ABCG2, SLC1A5 is overexpressed in many human tumors including breast cancers, and its overexpression has been correlated with poor prognosis (14, 15, 16, 17). We now show that ABCG2 regulates SLC1A5-mediated glutamine transport, accompanied by rewiring of cancer cell metabolism, enhanced autophagy, and increased tumor cell survival under stress conditions (18, 19, 20, 21). This observation of a role for ABCG2 in the control of glutamine metabolism is novel and may have implications for both normal cells in which ABCG2 is highly expressed as well as in diseases where ABCG2 is aberrantly overexpressed.

## Results

### The solute transporter SLC1A5 is present in an ABCG2 protein complex

To further explore the mechanism(s) whereby ABCG2 confers survival under stress conditions, we considered the possibility that ABCG2 may accomplish this through interactions with other membrane proteins. Given our recent observation that ABCG2 expression is associated with poor prognosis in patients harboring luminal A subtype breast tumors (TCGA data analysis, unpublished), we utilized the luminal breast adenocarcinoma cell line MCF-7 (very low ABCG2 expression) and its mitoxantrone (MX)-derived, ABCG2-overexpressing subline MCF-7/MX to perform the study. Data from additional cell lines are shown in Supporting Information.

**Figure 1:**
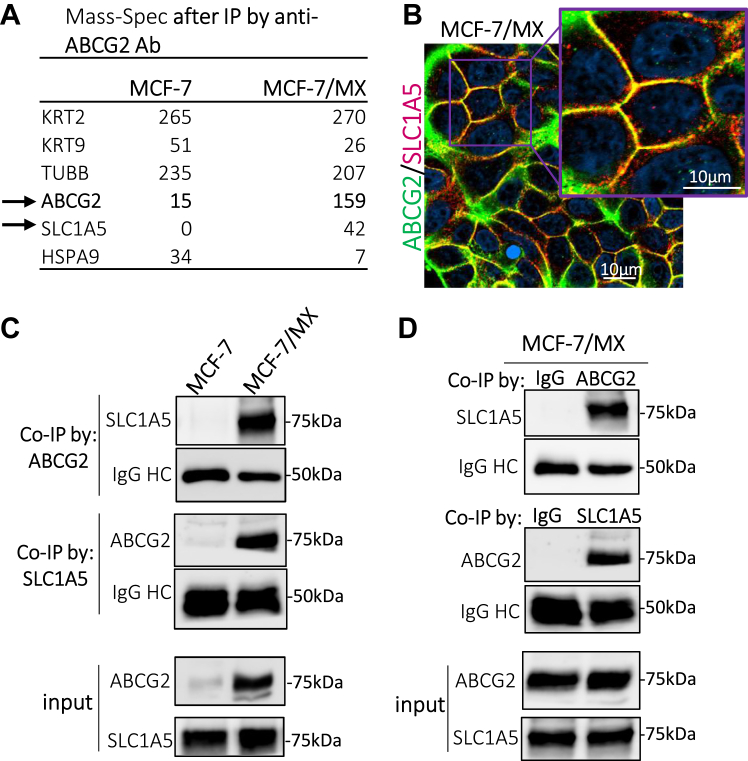
**The solute transporter SLC1A5 is present in a complex with the ABC transporter ABCG2 protein complex.***A*, representative proteins identified in an ABCG2 co-immunoprecipitation (co-IP) assay. *B*, immunofluorescence imaging of both ABCG2 (*green*) and SLC1A5 (*red*) on fixed MCF-7/MX cells. Co-localization of ABCG2 and SLC1A5 (*yellow*) is observed in discreet domains of the plasma membrane. *C*, Western blot analysis following co-IP using either an ABCG2 (*upper panel*) or SLC1A5 (*middle panel*) antibody on lysates of MCF-7 and MCF-7/MX cells demonstrated reciprocal pull-down. Note that SLC1A5 was easily detectable in co-IP complexes from ABCG2-overexpressing MCF-7/MX cells but not in low ABCG2-expressing MCF-7 cells, despite similar levels of SLC1A5 protein in both cell lines (*lower panel*). *D*, control for 1C, using either anti-mouse or anti-rabbit IgG antibody to verify the specificity of ABCG2 and SLC1A5 antibodies.

To begin these studies, lysates from MCF-7 and MCF-7/MX cells were immunoprecipitated with an ABCG2 antibody and then subject to mass spectrometry. Results from the two cell lines were compared and candidate ABCG2 binding partners were identified as those proteins primarily or exclusively found in the complexes isolated from MCF-7/MX cells. Notable among the candidate proteins was the neutral amino acid transporter SLC1A5 (ASCT2), the primary glutamine transporter in tumor cells. As shown in Figure 1*A* (arrow), considerable interaction among these two proteins was observed in ABCG2-overexpressing MCF-7/MX cells (42 units) relative to MCF-7 cells (0 units). This interaction was also detected in MCF-7/MX cells by co-immunofluorescence (Fig. 1*B*); while both ABCG2 (green) and SLC1A5 (red) existed independently within the membrane, significant colocalization of a subset of each protein was also observed (yellow). Similar co-localization was observed in the human choriocarcinoma cell line BeWo that intrinsically overexpresses ABCG2 (Fig. S1*C*).

The ABCG2-SLC1A5 interaction was confirmed by co-immunoprecipitation (co-IP) using either an ABCG2 or SLC1A5-specific antibody followed by western blotting. Consistent with our initial observations, SLC1A5 was easily detected within the ABCG2-IP complex in MCF-7/MX cells but not in MCF-7 cells (Fig. 1*C*, upper panel) as would be expected given the difference in ABCG2 levels in these two cell lines. Similarly, immunoprecipitation with a SLC1A5 antibody pulled down a significant amount of ABCG2 in MCF-7/MX cells but not in MCF-7 cells (Fig. 1*C*, middle panel). Protein input is shown in Figure 1*C*, lower panel. Notably, control co-IP using either rabbit IgG to replace the SLC1A5 antibody or mouse IgG to replace the ABCG2 antibody did not pull down corresponding partners (Fig. 1*D*, upper and middle panels, respectively) verifying the specificity of the assay. Similar results were observed when comparing HEK293 (immortalized kidney epithelial cells) with HEK293 cells stably transfected with an ABCG2 expression vector (Fig. S1*A*) and the lung carcinoma line H460 with its mitoxantrone selected subline H460/MX (Fig. S1*B*), indicating that this observation is not unique to the MCF-7 cell lines. Together, these data support the hypothesis that ABCG2 interacts, directly or indirectly, with the amino acid transporter SLC1A5. To our knowledge, this is the first observation of a physical interaction between members of these two transporter superfamilies, which led us to question whether this interaction has functional significance.

### ABCG2 increases SLC1A5-mediated glutamine uptake

**Figure 2:**
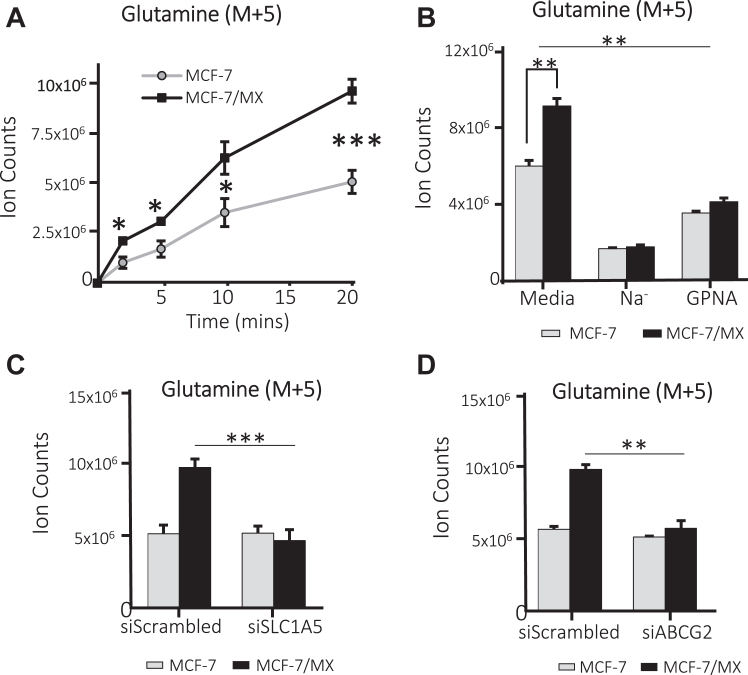
**ABCG2 regulates SLC1A5-mediated glutamine uptake.** [U-^13^C_5_]-glutamine tracer was added to culture medium and intracellular levels of glutamine (Glutamine (M + 5) were detected by LC/MS. Each data point represents at least 3 replicates. *A*, Gln uptake in MCF-7/MX cells was significantly higher over time than that detected in MCF-7 cells. *B*, inhibition of SLC1A5 transport by sodium depletion or use of the selective inhibitor GPNA markedly reduced Gln uptake and minimized the difference in uptake between MCF-7 and MCF-7/MX cells. *C* and *D*, knockdown of either SLC1A5 (*C*) or ABCG2 (*D*) also reduced the difference in glutamine uptake in MCF7/MX cells, confirming the role of both proteins in enhanced uptake in the ABCG2-overexpressing cells. ∗ Indicates *p* < 0.05; ∗∗ indicates *p* < 0.01; ∗∗∗ indicates *p* < 0.001.

SLC1A5 is the primary transporter for the conditionally essential amino acid glutamine; knockdown of SLC1A5 was shown to result in a 60% decrease in glutamine uptake in some cancer cells (22, 23). Given the interaction between ABCG2 and SLC1A5, we sought to determine whether ABCG2 impacts glutamine uptake. MCF-7 cell lines were incubated in a complete medium supplemented with the tracer [U-^13^C_5_]-glutamine at 37 °C for 1 to 20 min to allow probe uptake. Intracellular levels of ^13^C-glutamine (glutamine (M + 5)) were then measured using mass spectrometry (LC/MS) for isotope-labeled metabolites. As shown in Figure 2*A*, uptake of glutamine (M + 5) was 2-fold greater in MCF-7/MX cells as compared to MCF-7 cells at 20 min. Similar results were observed in HEK293/ABCG2 cells as compared to HEK293/vector controls (Fig. S2*A*), and in BeWo cells as compared to their ABCG2 knockout cell line 4B (Fig. S2, *B* and *C*).

As ABCG2 is not a glutamine transporter, the most likely explanation for these results was that ABCG2 was regulating glutamine uptake by another glutamine transporter, *i.e.*, SLC1A5. However, although SLC1A5 is the major glutamine transporter, with high affinity and high capacity observed in many cancer types (24, 25), other members of the solute carrier superfamily including SLC7A5 (LAT1) and SLC38A1/2 (SNAT1/2) have also been linked to tumor-specific glutamine uptake (26). To verify that the ABCG2-enhanced glutamine uptake was indeed dependent on SLC1A5, we took advantage of the fact that the activity of SLC1A5 and SLC38 requires Na^+^, while the activity of SLC7A5 is Na^+^-independent, providing an opportunity to narrow down the transporters that enable ABCG2-enhanced glutamine uptake. MCF-7 and MCF-7/MX cells were incubated in Na^+^-depleted medium prior to analysis of [U-^13^C_5_]-glutamine uptake, with the assumption that SLC1A5 and SLC38A1/2 function would be diminished in the absence of their key Na^+^ cofactor. As shown in Figure 2*B*, while MCF-7/MX cells exhibited significantly higher (∼30%) glutamine uptake compared to MCF-7 cells when incubated in Na+-replete medium, removal of Na+ from the media resulted in a marked (2∼3-fold) reduction in glutamine uptake in both cell lines, and eliminated the difference in uptake between the cell lines that were observed in Na+-replete medium. Therefore, SLC7A5 was eliminated from further consideration. To distinguish the contributions of SLC1A5 and SLC38A1/2 to the observed phenotype, cells were pre-treated with L-γ-glutamyl-p-nitroanilide (GPNA), a SLC1A5-specific inhibitor (27, 28, 29); marked reduction in glutamine uptake was observed, indicating that SLC1A5 was the major contributor to the increase in glutamine in MCF-7/MX cells. Finally, when the expression of either SLC1A5 (Fig. 2*C*) or ABCG2 (Fig. 2*D*) was knocked down using gene-specific siRNA, glutamine uptake in MCF-7/MX decreased to levels comparable to those seen in MCF-7 cells. Together, these data indicate that both SLC1A5 and ABCG2 are required for the increased uptake of glutamine in ABCG2-expressing cells. Given that both MCF-7 and MCF-7/MX express similar levels of SLC1A5 protein (Fig. 1*C*, input), the ABCG2-associated increase in glutamine uptake in MCF-7/MX strongly suggests that ABCG2 enhances the glutamine transport activity of SLC1A5.

### ABCG2 overexpression impacts tumor cell metabolism

**Figure 3:**
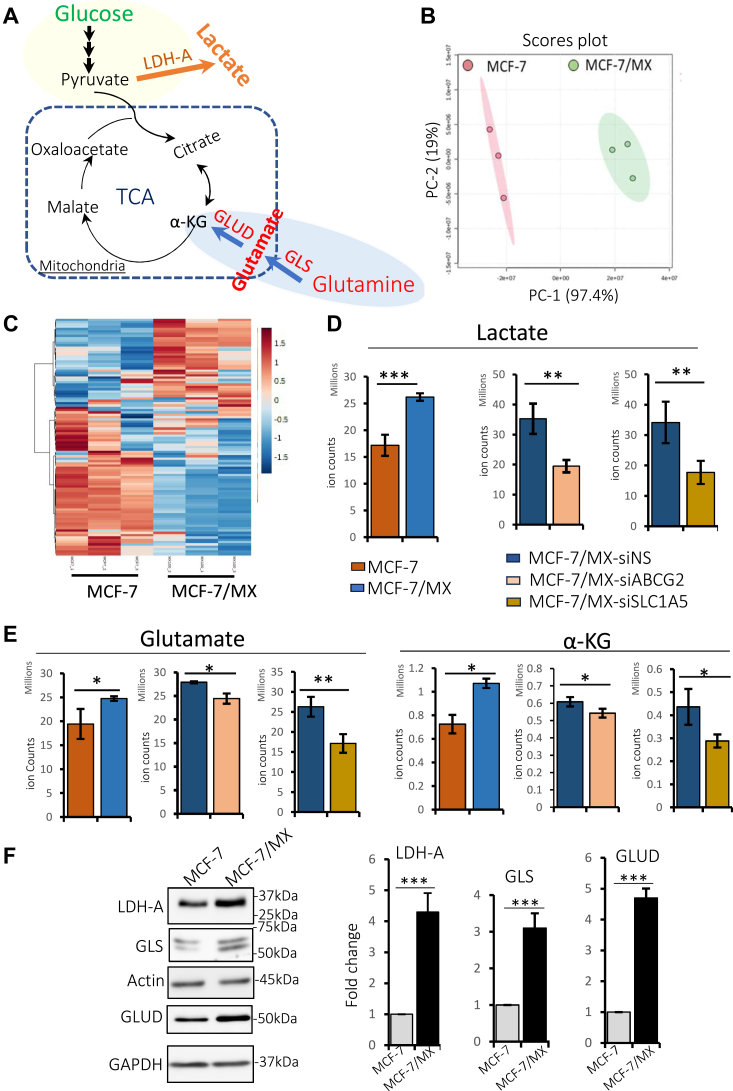
**ABCG2 overexpression impacts tumor cell metabolism.***A*, a schematic illustration of reprogrammed glucose metabolism (the Warburg effect) and altered glutamine metabolism (increased glutaminolysis) in cancer cells. *B*, principal component analysis revealed distinct metabolic profiles in MCF-7 and MCF-7/MX cells. Data of three replicate analyses are shown. *C*, clustering heatmap analysis revealed different levels of metabolites in MCF-7 and MCF-7/MX cells. Data of three replicate analyses are shown. Level difference ranges from −1.5-fold (*darkest blue*) to +1.5-fold (*darkest red*). *D*, steady-state analysis of MCF-7/MX metabolites revealed a ∼50% increase in lactate levels as compared to MCF-7 cells (*left graph*). Knockdown of either ABCG2 (*middle graph*) or SLC1A5 (*right graph*) in MCF-7/MX cells decreased the levels of lactate by ∼50%. *E*, steady-state analysis of MCF-7/MX metabolites revealed higher levels of glutamate and α-KG than those detected in MCF-7 cells (*left panel* for each metabolite); knockdown of either ABCG2 (*middle panel*) or SLC1A5 (*right panel*) in MCF-7/MX cells decreased the levels of both metabolites. *F*, Western blot analysis demonstrated an increase in levels of key enzymes involved in the Warburg effect and glutaminolysis in MCF-7/MX cells as compared to MCF-7/MX cells: LDH-A (Lactate dehydrogenase-A) mediates the conversion of pyruvate to lactate; GLS (Glutaminase) is the rate-limiting enzyme mediating conversion of glutamine to glutamate, GLUD (Glutamate dehydrogenase 1) is responsible for converting glutamate to α-KG. Actin and GAPDH served as loading controls. Experiments were repeated at least three times. ∗ indicates *p* < 0.05; ∗∗ indicates *p* < 0.01; ∗∗∗ indicates *p* < 0.001.

Metabolic reprogramming is a hallmark of cancer cells and is characterized by a dramatic shift of glucose-derived carbon from the mitochondrial TCA cycle to lactate production (the Warburg effect). This shift is often compensated by an increase in glutaminolysis, evidenced by increased glutamine uptake and conversion of glutamine to glutamate via glutaminase (GSL), followed by increased conversion of glutamate to α-ketoglutarate (α-KG) via glutamate dehydrogenase (GLUD)) to refuel the TCA cycle (Fig. 3*A*) (30). To determine whether ABCG2-overexpressing cells had undergone metabolic reprogramming, we performed metabolomics analyses on MCF-7 and MCF-7/MX cells. Initial assessments by principal component analysis (PCA) (Fig. 3*B*) and a clustering heatmap (Fig. 3*C*) of the top 25% differentially expressed metabolites revealed distinct profiles between the two cell lines. Notably, MCF-7/MX cells exhibited a greater degree of Warburg effect compared to MCF-7 cells as evidenced by the marked increase (almost 2-fold) in the production of lactate (Fig. 3*D*, left). When either ABCG2 or SLC1A5 was knocked down in MCF-7/MX cells, lactate production decreased by ∼50% (Fig. 3*D*, middle and right, respectively), indicating that both ABCG2 and SLC1A5 contributed to the increased Warburg effect. Moreover, higher levels of glutamate and α-KG were detected in MCF-7/MX compared to MCF-7 cells, and knockdown of either ABCG2 or SLC1A5 lowered the levels of both metabolites in MCF-7/MX cells (Fig. 3*E*), suggesting their involvement in increased glutaminolysis in ABCG2-overexpressing cells. Importantly, key enzymes that mediate the Warburg effect (LDH-A) and glutaminolysis (GLS and GLUD) were more highly expressed in MCF-7/MX versus MCF-7 cells (Fig. 3*F*), strongly supporting the notion that these ABCG2-overexpressing cells have reprogrammed major metabolic pathways, including those involved in glutamine metabolism.

**Figure 4:**
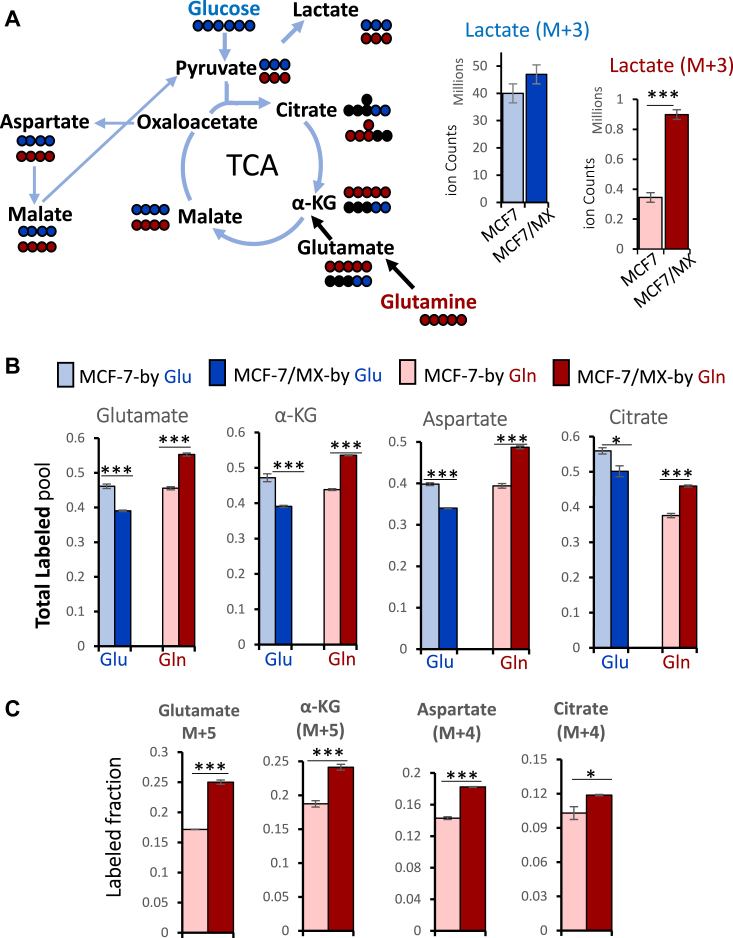
**Glutamine contributes to reprogrammed metabolism in ABCG2-overexpressing cells.** Metabolomic analyses were performed using either [U^13^C_6_]-Glucose tracer (*blue*, Glu) or [U^13^C_5_]-Glutamine tracer (*red*, Gln). *A*, *left* - Pathways of lactate production. *Dots* indicate the carbons derived from either glucose (*blue*) or glutamine (*red*). *Right* - Higher contributions from glutamine to lactate were observed in MCF-7/MX versus MCF-7 cells, while the contribution from glucose to lactate was not significantly different. ∗∗∗ indicates *p* < 0.001. *B*, more of the major TCA intermediates, including glutamate, α-KG, aspartate, and citrate, were derived from glutamine metabolism in MCF-7/MX cells (*dark red bars*) as compared to MCF-7 cells (*light red bars*), while the contribution of glucose was lower in MCF-7/MX cells than in MCF-7 cells (*dark blue versus light blue bars*). *C*, the MCF-7/MX cells had higher levels of glutamine tracer-labeled isotopomers directly derived from glutamine anaplerosis when compared to MCF-7 cells.

To further evaluate the contribution of glutamine to the metabolic reprogramming of MCF-7/MX cells, analyses were performed using the tracers [U^13^C_6_]-glucose and [U^13^C_5_]-glutamine, both of which can be catabolized to TCA intermediates as well as to lactate (M + 3) (see Fig. 4*A*, diagram). As shown in Figure 4*A* (graphs), glucose (blue bars) is the primary source of lactate production as expected, with a fairly equal contribution in MCF-7 and MCF-7/MX cells. In contrast, the contribution of glutamine (red bars) to lactate production was more than doubled in MCF-7/MX cells relative to MCF-7 cells. Moreover, the contribution of glutamine to TCA intermediates (such as glutamate, αKG, aspartate, and citrate) was higher in MCF-7/MX cells (Fig. 4*B*, dark red bars) relative to MCF-7 (light red bars), while the contribution from glucose to these metabolites was lower in MCF-7/MX cells (dark blue bars) as compared to MCF-7 (light blue bars). Finally, MCF-7/MX cells exhibited increased levels of metabolites produced via glutaminolysis (glutamate (M + 5) and αKG (M + 5)), and glutamine anaplerosis (aspartate (M + 4) and citrate (M + 4)) (Fig. 4*C*, labeled fraction). Similar results were observed in BeWo cells when compared to ABCG2 knockout line 4B (Figs. S3 and S4). Taken together, these data further support the notion that the ABCG2-overexpressing MCF-7/MX cells have reprogrammed metabolism with increased glutaminolysis and glutamine anaplerosis.

### ABCG2-overexpressing cells are more resilient to oxidative stress

Glutamine anaplerosis is often enhanced in tumor cells to replenish TCA cycle intermediates needed to support the high demand for building blocks such as proteins, lipids and nucleic acids, as well as for energy and redox homeostasis. Glutamine has also been implicated in sustaining redox balance to protect cells from oxidative stress (23). This need is largely accomplished through two outcomes of glutamine metabolism (1): glutamine-derived TCA intermediates necessary to support a robust production of NADPH (31, 32, 33) and macromolecules, and (2) glutamine-derived glutamate to be utilized as a direct substrate for glutathione (GSH) biosynthesis.

**Figure 5:**
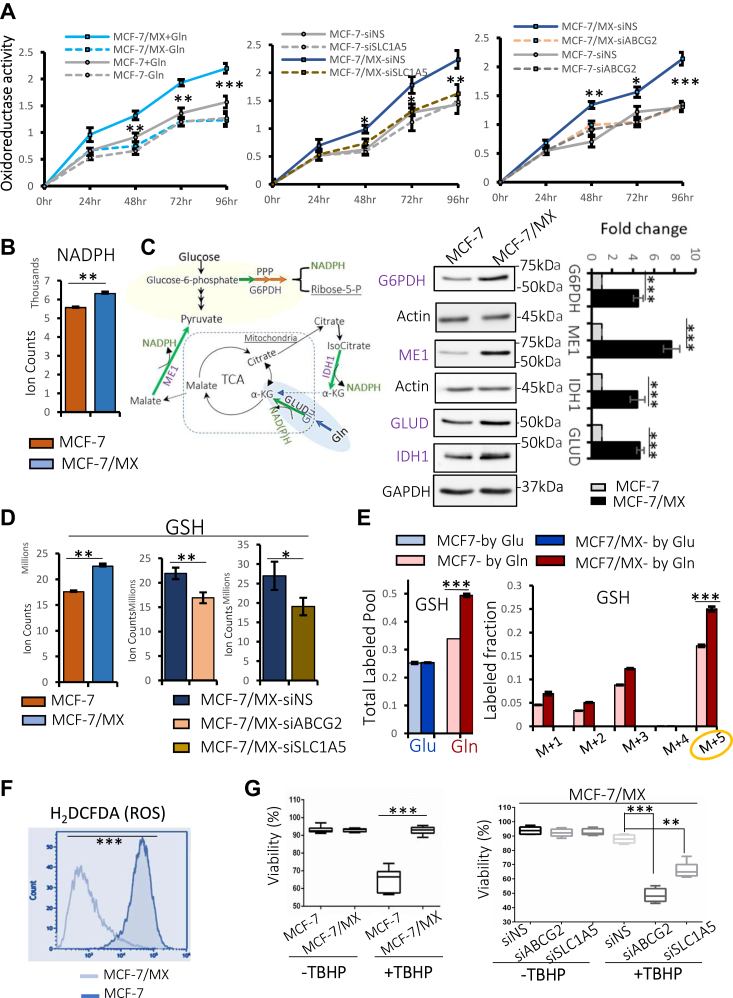
**ABCG2-overexpressing cells are more resilient in the face of oxidative stress.***A*, oxidoreductase activity was assessed in MCF-7 and MCF-7/MX cells in the presence or absence of Gln (*left graph*), Oxidoreductase activity assayed in both cell lines following knockdown of SLC1A5 (*middle graph*) and ABCG2 (*right graph*). Either depletion of Gln or knockdown of SLC1A5 or ABCG2 led to decreased oxidoreductase activity in MCF-7/MX cells to levels similar to that in MCF-7. Each data point represents results from 8 replicates. ∗ indicates *p* < 0.05; ∗∗ *p* < 0.01. ∗∗∗ *p* < 0.001. *B*, analysis of steady-state metabolome profiles revealed higher levels of NADPH in MCF-7/MX cells compared to MCF-7 cells. *C*, *left* - schematic showing primary pathways/reactions responsible for NADPH generation. *Right*—Western blot analyses demonstrated increased expression of key enzymes mediating NADPH production. Actin and GAPDH served as loading controls. Experiments were repeated at least three times. *D*, higher levels of GSH were observed in MCF-7/MX cells compared to MCF-7; knockdown of either ABCG2 or SLC1A5 in MCF-7/MX cells decreased the levels of GSH. *E*, *left*—[U^13^C_6_]-Glucose and [U^13^C_5_]-Glutamine tracer analyses revealed significantly higher contribution from glutamine to GSH in MCF-7/MX cells (*dark red bar*) compared to MCF-7 cells (*light red bar*), while no difference was observed in the contribution of glucose between these two cell lines (*blue bars*); *Right* - M + 5 is the major form of glutamine-derived GSH in MCF-7/MX cells, suggesting a direct role of glutamine in GSH biosynthesis. *F*, lower levels of ROS were detected in MCF-7/MX cells as compared to MCF-7 cells when analyzed by H_2_DCFDA flow cytometry. *G*, *left*—MCF-7/MX cells remained viable following a 24-h induction of oxidative stress with tert-Butyl hydroperoxide (TBHP) while significant cell death was observed in MCF-7 cells. *Right*—MCF-7/MX cells were more vulnerable to TBHP-induced ROS when either ABCG2 or SLC1A5 was knocked down. Each assay was performed in triplicate and repeated at least three times. ∗ indicates *p* < 0.05; ∗∗ *p* < 0.01. ∗∗∗ *p* < 0.001.

NAD(P)H-dependent oxidoreductases, which catalyze the reduction or oxidation, respectively, of NAD(P)H and NAD(P), are responsible for the catalysis of multiple redox reactions in the cell. As such, they are pivotal players in many key metabolic processes and can be used as an indirect marker of metabolic activity associated with NAD(P)H status. To begin to query a role for ABCG2/SLC1A5-enhanced glutaminolysis in redox homeostasis, the activity of these oxidoreductases was measured. As shown in Figure 5*A*, MCF-7/MX cells contained higher levels of dehydrogenase activity when compared to MCF-7 cells (left graph, solid blue *versus* solid gray line). Notably, depletion of glutamine decreased the levels to those observed in MCF-7 cells (dotted blue *versus* dotted gray line). Similar results were obtained following the knockdown of either SLC1A5 (middle graph) or ABCG2 (right graph), implicating these transporters in the enhanced oxidoreductase activity observed in MCF-7/MX cells.

Consistent with these initial experiments, MCF-7/MX cells were observed to contain higher levels of NADPH (Fig. 5*B*) as well as key enzymes responsible for NADPH production (Fig. 5*C*), including G6PDH (a rate-limiting enzyme in the pentose phosphate pathway (PPP)), ME1 (responsible for converting shuttled malate into pyruvate), IDH1(cytosolic isocitrate dehydrogenase), and GLUD (glutamate dehydrogenase that converts glutamate into α-KG). Notably, MCF-7/MX cells also contained higher concentrations of GSH, which were decreased when either ABCG2 or SLC1A5 was knocked down (Fig. 5*D*). The increased GSH was directly derived from glutamine, as demonstrated by increased counts of total pools labeled with glutamine tracer in MCF-7/MX compared to that in MCF-7 (Fig. 5*E*, left, dark red *versus* light red) and by analysis of individually labeled fractions, especially GSH (M + 5) (Fig. 5*E*, right graph).

Taken together, these results suggest that ABCG2-overexpressing cells could be more resistant to oxidative stress than their low-expressing counterparts. To test this hypothesis, intracellular ROS (reactive oxygen species) was measured using the H_2_DCFDA flow cytometry method. As shown in Figure 5*F*, strikingly lower levels of ROS were detected in MCF-7/MX cells as compared to MCF-7 cells, suggesting that even in the absence of additional stress ABCG2 overexpressing cells are better able to suppress ROS. Moreover, when subjected to TBHP-induced ROS (Fig. 5*G*, left panel) MCF-7/MX cells were markedly more resilient than MCF-7 cells, evidenced by much higher viability. Notably, MCF-7/MX cells were far more vulnerable to ROS when either ABCG2 or SLC1A5 was knocked down (Fig. 5*G*, right panel), consistent with the hypothesis that the ABCG2-regulated, SLC1A5 -mediated increase in glutamine metabolism conferred a survival advantage in the face of oxidative stress. Similar results were observed in other ABCG2-overexpressing cells, including the human placental choriocarcinoma BeWo cells that have high levels of ABCG2 expression (Fig. S5, *A*–*C*).

### Increased SLC1A5-mediated glutamine uptake is required for ABCG2-enhanced autophagy

**Figure 6:**
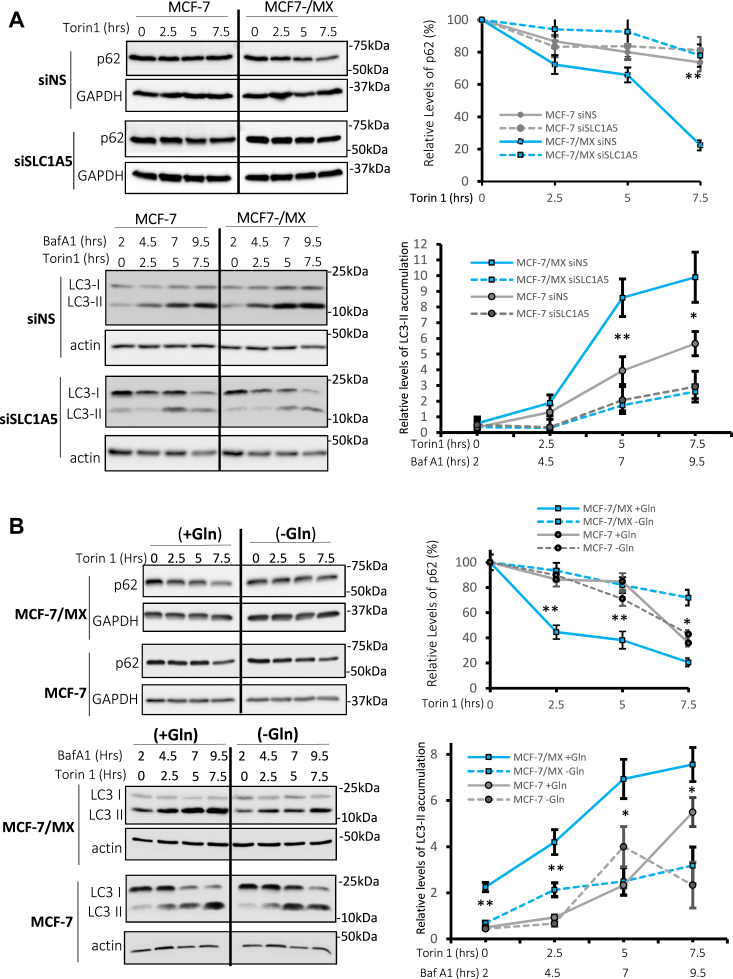
**Increased SLC1A5-mediated glutamine uptake is required for ABCG2-enhanced autophagy flux.***A*, *top panels*—Western blot analysis of p62 following induction of autophagy by Torin 1. *Lower panels* - Western blot analyses of LC3-II accumulation in the presence of Baf A1 following Torin 1 treatment. As evidenced by the relative rates of p62 degradation and LC3-II accumulation, siRNA knockdown of SLC1A5 reduced the rate of autophagy flux in MCF-7/MX cells (*dotted versus solid blue line*) to levels similar to those in MCF-7 cells (*grey lines*). siNS were used as a control. *B*, *top panels*—Western blot analysis of p62 following induction of autophagy by Torin 1 in cells cultured with (+Gln) or without glutamine (-Gln). While MCF-7/MX cells exhibited a faster rate of autophagy flux (indicated by p62 degradation, *solid blue lines*), glutamine depletion markedly reduced this rate (*dotted blue lines*) to levels similar to what was observed in MCF-7 cells (*solid grey lines*). *Lower panels* – Western blot analyses of LC3-II accumulation in the presence of Baf A1 following Torin 1 treatment. A greater rate of accumulation of LC3-II was observed in MCF-7/MX cells compared to MCF-7 cells in the presence of glutamine, while accumulation decreased to the levels similar to MCF-7 in the absence of exogenous glutamine. GAPDH and actin were used as loading controls. ∗ indicates *p* < 0.05; ∗∗ *p* < 0.01.

Our lab and others have previously shown that ABCG2 overexpression augments autophagy flux. As the mechanism by which this occurs has not yet been determined, we considered the possible involvement of SLC1A5 in this phenotype. To test this hypothesis, SLC1A5 was knocked down by RNAi in MCF-7 and MCF-7/MX cells and autophagy was induced by the TORC1 inhibitor Torin 1. As shown in Figure 6*A* (upper panel), downregulation of SLC1A5 resulted in a marked decrease in p62 degradation in MCF-7/MX cells (compare solid and dotted blue lines) with little change observed in MCF7 cells (compare solid and dotted gray lines). Consistent with this observation, when cells were pre-treated with the lysosomal inhibitor Bafilomycin 1 (Baf A1) and autophagy induced with Torin 1, knockdown of SLC1A5 resulted in reduced LC3-II accumulation in MCF-7/MX cells to a level similar to what was observed in MCF-7 cells (Fig. 6*A*, lower panel, solid blue *versus* dotted blue and gray lines). To verify that it is the glutamine uptake activity of SLC1A5 that is involved in this phenotype, cells were grown in the presence or absence of glutamine and Torin 1-induced autophagy was assessed. As shown in Figure 6*B*, the removal of glutamine from the media decreased the rate of p62 degradation (upper panel) and LC3-II accumulation (lower panel) in MCF-7/MX cells (blue lines) with little effect on MCF-7 cells (gray lines), confirming a role for SLC1A5-mediated glutamine uptake in ABCG2-enhanced autophagy flux.

## Discussion

ABCG2 is well known for its ability to efflux a variety of chemotherapeutic agents, thereby conferring MDR on tumor cells in which it is overexpressed. However, several studies have suggested that ABCG2 can promote tumor survival via MDR-independent mechanisms (5, 6, 7, 8). Indeed, a number of studies have shown that ABCG2 can protect cancer cells against stressors such as radiation, a function that cannot be explained by its role as a drug transporter (5, 6, 7, 8, 34). In the present study we show that, in addition to its chemoprotective role in cancer cells, ABCG2 can regulate cancer cell metabolism via regulation of the glutamine transporter SLC1A5, resulting in increased glutaminolysis and enhanced redox regulation.

The observation that ABCG2 interacts with the amino acid transporter SLC1A5 to regulate glutamine metabolism suggests a new and previously unexplored role for ABCG2 in the regulation of cancer cell survival. Glutamine dependency is a hallmark of cancer cell metabolism; both *in vitro* and *in vivo* studies have shown that different tumor cells may vary considerably in their demands for exogenous glutamine (35, 36). With significantly enhanced aerobic glycolysis re-routing glucose-supplied carbon away from mitochondria, many tumors rely on glutamine-mediated TCA anaplerosis to fulfill the high demands placed on mitochondria to supplement precursors of cellular building blocks, produce reducing equivalents (NADPH/GSH), and generate ATP (30). Indeed, mitochondria-derived ATP has been suggested to be the major source of energy to fuel ABC transporter efflux (37), indicating that increased activity of ABCG2 resulted in a higher demand for mitochondrial oxidative phosphorylation (OXOPH), one of the major sources of internal ROS. Interestingly, increased expression of several key enzymes involved in NADPH production was observed in ABCG2-overexpressing MCF-7/MX cells compared to parental MCF-7 cells, a potential adaptive response to mitigate their higher levels of ROS. Consistent with this observation, higher levels of reducing equivalents (NADPH/GSH) were observed in MCF-7/MX cells and they were more resilient to oxidative stress, in line with the notion that glutamine metabolism plays a major role in maintaining cancer cell redox homeostasis (38) and in agreement with previous studies by our lab (3) and others (39) showing that ABCG2 protects against inducers of oxidative stress including radiation and nutrient deprivation. Moreover, it is intriguing to speculate that a well-buffered redox environment promoted by ABCG2-regulated SLC1A5 activity may prevent certain cysteine residues of SLC1A5 from oxidative modifications that reduces its transport activity, thereby maintaining increased SLC1A5-mediated glutamine uptake (40). Thus, ABCG2-dependent augmentation of glutamine uptake and glutaminolysis could enhance survival of cancer cells subject to metabolic stress.

Our data implicating SLC1A5-mediated uptake of glutamine in increased autophagy flux in ABCG2-overexpressing cells is consistent with several prior studies showing that increased glutamine can promote autophagy under both basal and stressed conditions (20, 41, 42, 43). However, the effect of glutamine on autophagy is complex; indeed, crosstalk between glutamine metabolism and autophagy has been shown to vary across different tumor types (18, 19, 20, 21, 44). An example of this can be found in a recent publication by P. Nicklin, *et al.* (45), where downregulation of SLC1A5 led to an increase in autophagy. In this study HeLa cells, which are highly dependent on exogenous glutamine, were first starved to decrease activation of mTOR, thereby activating autophagy. When exogenous glutamine and essential amino acids were added back to the media, the glutamine imported by SLC1A5 was effluxed in exchange for the uptake of exogenous leucine by another antiporter, which in turn activated mTOR and inhibited autophagy. Subsequent downregulation of SLC1A5 reduced leucine uptake, preventing reactivation of mTOR and thus inhibition of autophagy. In contrast, the MCF-7 cell pairs used in this study, which are able to synthesize sufficient levels of glutamine to maintain mTOR activation, were grown in complete medium. Under these conditions, exogenous glutamine imported by SLC1A5 was shuttled to the TCA cycle and glutaminolysis in ABCG2-overexpressing cells. Therefore, downregulation of SLC1A5 in these cells would be expected to have a markedly different impact on autophagy, given the potential impact from metabolites of glutaminolysis that may activate autophagy, such as ammonia (46, 47). This suggests that the metabolic phenotype of a given tumor cell, its relative dependency on exogenous glutamine, and the ultimate fate of exogenous glutamine is likely to influence the impact of changes in glutamine uptake on autophagy.

The mechanism by which ABCG2 interacts with and regulates glutamine uptake via SLC1A5 has yet to be determined. Several ABC transporters, including those involved in the MDR phenotype, have been shown to interact with other proteins (ABCA1 and B2-syntrophin (48); ABCB1 and CD44 (49, 50); ABCC4 and CFTR (51). However, to our knowledge, this is the first observation of a functionally significant physical interaction between members of the ABC and Solute Carrier (SLC) superfamilies of transporters and opens the way for investigations into other ABC/SLC transporter functional interactions. Current studies are underway to determine whether ABCG2 and SLC1A5 interact directly or as part of a larger complex; either way, this interaction is accompanied by an altered metabolic profile in ABCG2-overexpressing cells. It is interesting to note that it has long been speculated, and in many cases demonstrated, that ABC transporters play a role in tumor cell survival, proliferation, and metastasis that is MDR-independent (34). Most studies querying the mechanism by which this occurs have focused on the known endogenous substrates of the ABC transporter as regulators of the altered phenotype. Our study suggests an additional possibility - that ABC transporters may impact cellular function indirectly through the regulation of other membrane transporters, potentially controlling both the uptake and metabolism of their key nutrient substrates. Indeed, this may also explain some of the observations linking ATP transporters to metabolic disease (52).

In summary, we have identified a novel role for ABCG2 in cancer cell metabolism. Whether this regulation occurs *in vivo* is currently under investigation. Nevertheless, as both ABCG2 (53, 54, 55) and SLC1A5 (14, 15, 16) have been found overexpressed in various cancers, and anti-cancer drugs targeting SLC1A5/glutamine metabolism have been in development (56), it is feasible that further studies may inform novel combination therapies for tumors overexpressing these two membrane proteins.

## Experimental procedures

### Cell culture and viability assays

The human breast adenocarcinoma cell line MCF-7 and its mitoxantrone (MX)-derived ABCG2-overexpressing multidrug-resistant subline MCF-7/MX were kindly provided by Dr Susan Bates, National Institutes of Health, MD. Both MCF-7 lines were grown in an improved minimum essential medium (IMEM, ThermoFisher, A1048901) supplemented with 10% FBS (fetal bovine serum). MCF-7/MX cells were maintained in the presence of 100 nM MX (Sigma, M6545) (57). The human lung carcinoma cell lines H460 and H460/MX (Supplementary data) were also provided by Dr Bates and were maintained in RPMI-1640 medium (Gibco RPMI 1640, 31870017) supplemented with 2 mM L-glutamine and 10% FBS. H460/MX cells were maintained in the presence of 20 nM MX. HEK293 cells (ATCC CRL-1573) stably transfected with either pcDNA or pcDNA-ABCG2 (Supplementary data) were maintained in Dulbecco's modified Eagle's medium (DMEM, Cellgro, 15-017-CV) containing 10% FBS, 2 mM L-glutamine and 2 mg/ml geneticin (Invitrogen, 10131-035). The human placental choriocarcinoma cell line BeWo (ATTC CCL-98) and its ABCG2 KO subclone BeWo/4B (Supplementary data) were maintained in F-12K medium (ATCC, 30-2004) supplemented with 10% FBS. BeWo/4B was generated and verified by the Rutgers-CINJ Genome Editing Shared Resource Core Facility using CRISPR technology. All cells were regularly tested for *mycoplasma* using the PCR *Mycoplasma* Test Kit I/C (PromoCell, PK-CA91-1048). In general, cells were maintained in culture for not more than 15 passages. Cell line authentication was performed at the Rutgers Molecular Resource Facility using short tandem repeat (STR) profiling, and cell cultures were regularly monitored for phenotypic appearance and behavior.

Trypan blue exclusion assays were used to assess cell viability following the manufacturer’s instructions (LUNA^IITM^ Automated Cell Counter, Transilliminators). Cells were seeded at 2.0X10^5^ cells per well in 6-well cell culture plates. Each condition was performed in triplicate at least three times.

### Western blot analyses

Whole-cell lysates were collected in RIPA Buffer with the addition of protease inhibitors (Roche, #11836170001). Western blot assays were conducted as previously described (3) using mouse anti-ABCG2 monoclonal antibody (1:1000, Kamiya Biomedical Company, #MC-177), guinea pig anti-SQSTM1 (p62) monoclonal antibody (1:2000; Progen, #GP62-C), rabbit anti-SLC1A5 (ASCT2) antibody (1:1,000, Cell Signaling Technology, #5345), rabbit anti-GAPDH antibody (1:7000, Cell Signaling Technology, #2118), goat anti-actin antibody (1:500, Santa Cruz Biotechnology, sc-47778), rabbit anti-LDHA antibody (1:1000, Cell Signaling Technology, #2012), rabbit anti-GLS antibody (1:1000, Proteintech, #12855-1-AP), mouse anti-GLUD1/2 antibody (1:500, Santa Cruz Biotechnology, sc-515542), mouse anti-G6PDH antibody (1:500, Santa Cruz Biotechnology, sc-373886), mouse anti-ME1 antibody (1:500, Santa Cruz Biotechnology, sc-36589), and mouse anti-IDH1 antibody (1:500, Santa Cruz Biotechnology, sc-515396), followed by corresponding secondary antibodies from Santa Cruz Biotechnology (mouse anti-rabbit, sc-2357 and m-IgG BP-HRP, sc-516102). Immunoreactive proteins were visualized using an enhanced chemiluminescent system (SuperSignal West Pico Plus, ThermoFisher Scientific, #34577) following the manufacturer's recommendations. Images were acquired by Amersham Imager 680 (GE), while specific protein bands were analyzed by the Amersham Imager 680 analysis software and quantified using ImageJ software tools. Antibody specificity was verified according to manufacturer’s recommendations, either by (1) analyzing lysates from cells with specific gene knockdown or (2) detecting specific protein produced from recombinant DNA encoding genes.

### ABCG2 and SLC1A5 siRNA knockdown

Customized ON-Target plus ABCG2 siRNA, synthesized by ThermoFisher Scientific according to a previously identified sequence that targets exon 7 in ABCG2 mRNA (58), was used to downregulate ABCG2. SmartPool siGENOME specifically targeting human SLC1A5 (ThermoFisher Scientific) was used to knock down SLC1A5 expression. Cells were seeded at 3.5 × 10^5^ per well in 6-well plates in regular medium the day before transfection. Either gene-targeting siRNA (100 nM) or scrambled siRNA (100 nM) (siGENOME, non-targeting siRNA, ThermoFisher Scientific) was transfected into cells using Lipofectamine 3000 reagents (Life Technologies, US, #L3000008) following the manufacturer’s instruction. Cells were incubated for 48 to 72 h prior to specified treatment and analysis.

### ABCG2 interacting protein analysis

Approximately 1.0 × 10^6^ cells were collected in lysis buffer (50 mM Tris HCl, 150 mM NaCl, 1.0% (v/v) Triton X-100, 0.5% (w/v) sodium deoxycholate, 0.1% (w/v) SDS, 1 mM EDTA, I mM pyrophosphate, 10 mM glycerophosphate, 50 mM NaF, 0.5 mM orthovanadate) supplemented with EDTA-free protease inhibitors (Roche # 4693132001). Protein concentrations were determined using the BCA assay (Pierce BCA Protein Assay Kit, ThermoFisher Scientific, #23227). 1 mg of total protein was precleared for 1 h using protein G/A Sepharose beads (Invitrogen, #101242/#101041), then incubated with primary antibody (either anti-ABCG2 MXP-21 or anti-SLC1A5) at 4 °C overnight. Precipitation was performed the next day following the addition of protein G/A Sepharose beads and incubation was continued for 3 h at 4 °C prior to analysis by Western blot.

### Mass spectrometry analysis

Cell lysates were immunoprecipitated using the mouse anti-ABCG2 monoclonal antibody MXP-21 (Kamiya Biomedical, #MC-177) and immunoprecipitants were separated by SDS-PAGE followed by silver staining. Bands that were predominant in the MCF-7/MX cells relative to MCF-7 cells were excised and analyzed by peptide-based quantitative mass spectrometry at the Rutgers Biological Mass Spectrometry Core Facility.

### Immunofluorescence

Cells were grown on cover slips in 6-well plates, then fixed with 4% formaldehyde and subjected to immunofluorescence staining following the recommended protocol (Abcam), using primary antibodies mouse anti-ABCG2 (1:200; Kamiya Biomedical, #MC-177) or rabbit anti-SLC1A5 Ab (1:100; Cell Signaling, #5345). Either anti-mouse FITC-conjugated (1:200, Molecular Probes, A-11001) or anti-rabbit Rhodamine-conjugated secondary antibody (1:200, Molecular Probes, A-11009) was used, respectively. Images were acquired on a Nikon A1R-Si Confocal Microscope System housed at the CINJ Advanced Microscopy Shared Resource Core Facility.

### Metabolomics

Approximately 8.0 × 10^5^ cells were seeded in 60 cm^2^ dishes in triplicate and incubated for 18 h at 37 °C. Media was aspirated and cells were washed with warm PBS then lysed in 1 ml ice cold methanol:acetonitrile:water (40:40:20) with 0.5% formic acid. Plates were immediately transferred to ice and incubated for 5 min prior to the addition of 50 μl 15% NH_4_HCO_3_. Samples were analyzed at the Rutgers CINJ Metabolomic Shared Resource Core Facility, focusing on metabolites involved in glycolysis, glutaminolysis, and the TCA cycle. Data were analyzed using the MetaboAnalyst 5.0 (https://www.metaboanalyst.ca/home.xhtml).

For [U^13^C_5_]-glutamine uptake assays, cells were seeded in triplicate in 100 mm^2^ dishes for 18 h at 37 °C followed by the addition of [U^13^C_5_]-glutamine in place of regular glutamine for the time indicated (maximum time = 20 min). Samples were collected and analyzed as described above. For [U^13^C_5_]-glutamine and [U^13^C_6_]-glucose tracer metabolomics, cells were seeded in 60 mm^2^ dishes in triplicate with either [U^13^C_5_]-glutamine or [U^13^C_6_]-glucose replacing unlabeled glutamine or glucose in the medium. Following an 18 h incubation at 37 °C, samples were collected as described above for intracellular isotopomer analysis, focusing on glycolysis, glutaminolysis, and TCA cycle metabolites.

### Metabolism assay

NAD(P)H-dependent oxidoreductase activity was measured using the MTS assay kit as recommended by the manufacturer (Promega, #G3582). Cells were seeded at 7.0 × 10^3^ cells per well in 96-well cell culture plates with eight replicates for each condition. Reactive agents were added at the indicated time post-seeding and cells were incubated for an additional 2 h, followed by colorimetric spectrometry at 490 nm.

### Reactive oxygen species assay

Cells were seeded at 3.0 × 10^5^ cells per well in 6-well cell culture plates the day before the assay. Approximately 30 min before collection for flow cytometry, cells were washed with HEPES buffered salt solution (HBSS) and incubated at 37 °C in fresh medium with reduced (2%) FBS, no phenol red, and 10 μM freshly prepared H_2_DCFDA (Sigma, #287810). Following incubation, cells were collected by trypsinization, washed thoroughly with HBSS, and immediately analyzed for DCF signal by flow cytometry at the Rutgers Flow Cytometry Core Facility. Analysis of flow cytometric data was carried out using Kaluza Analysis Software (Beckman).

### Statistical analysis

All data are expressed as the mean ± SD. Statistical analyses were performed using either the MS Excel statistical package functions or GraphPad Prism V6. Two groups were compared using the two-tailed paired students *t* test. *p* values are expressed as follows: 0.05> *p* >0.01 as ∗; 0.01> *p* >0.001 as ∗∗*p* <0.001 as ∗∗∗. *p* values smaller than 0.05 were considered statistically significant.

## Data availability

Raw data is available from the corresponding author upon request.

## Supporting information

This article contains supporting information.

## Conflict of interest

The authors declare that they have no conflicts of interest with the contents of this article.
