# Supplementary material for: ABCG2 and SLC1A5 functionally interact to rewire metabolism and confer a survival advantage to cancer cells under oxidative stress
Source: J Biol Chem. 2024 Apr 18;300(6):107299. doi: 10.1016/j.jbc.2024.107299 (PMC11131071; doi:10.1016/j.jbc.2024.107299)
Supplement: Supporting Figures S1–S5 [file mmc1.pdf]

S1. The solute transporter SLC1A5 is present in an ABCG2 protein complex.

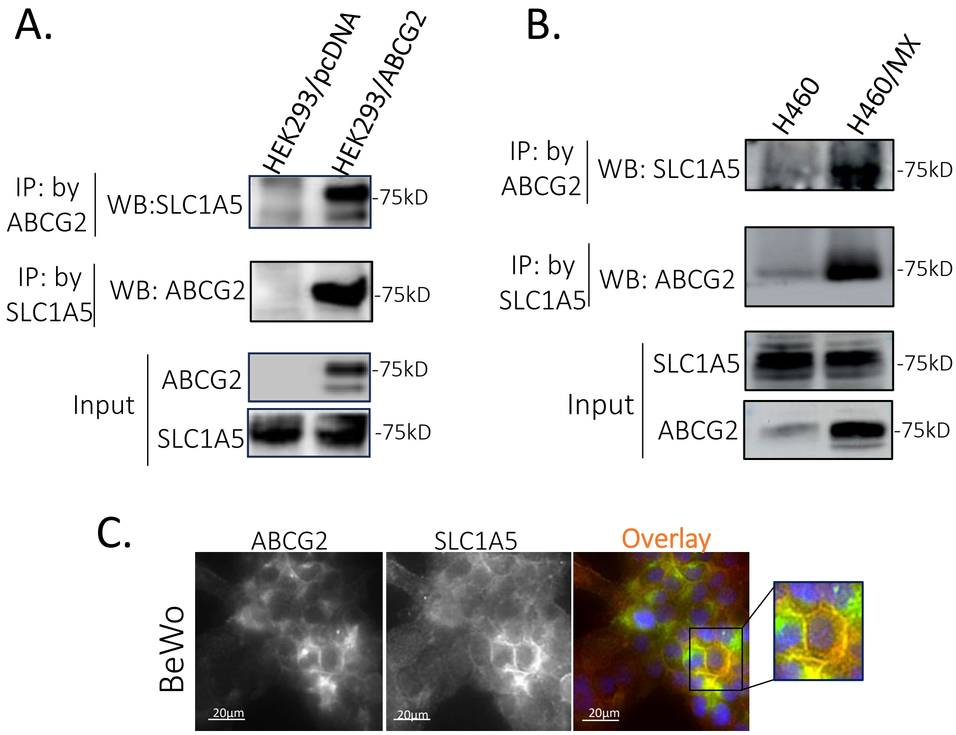

Fig S1. The solute transporter SLC1A5 is present in an ABCG2 protein complex. (A) Western blot analysis of SLC1A5:ABCG2 complexes in the human embryonic kidney cell line HEK293 and its ABCG2-transfected subline HEK293/ABCG2. Top panel - SLC1A5 co-immunoprecipitated with an ABCG2 antibody; middle panel - ABCG2 immunoprecipitated with an SLC1A5 antibody; lower panel - Input prior to immunoprecipitation. (**B**) Western blot analysis of SLC1A5:ABCG2 complexes in the human lung carcinoma cell line H460 and it ABCG2-overexpressing, mitoxantrone-selected subline H460/MX. Top panel - SLC1A5 co-immunoprecipitated with an ABCG2 antibody; middle panel - ABCG2 immunoprecipitated with an SLC1A5 antibody; lower panel - Input prior to immunoprecipitation. **(C)** Co-immunofluorescence staining of ABCG2 and SLC1A5 in the huma placental choriocarcinoma BeWo cells; image overlay shows co-localization of these two proteins on the membrane.

S2. ABCG2 regulates SLC1A5-mediated glutamine uptake

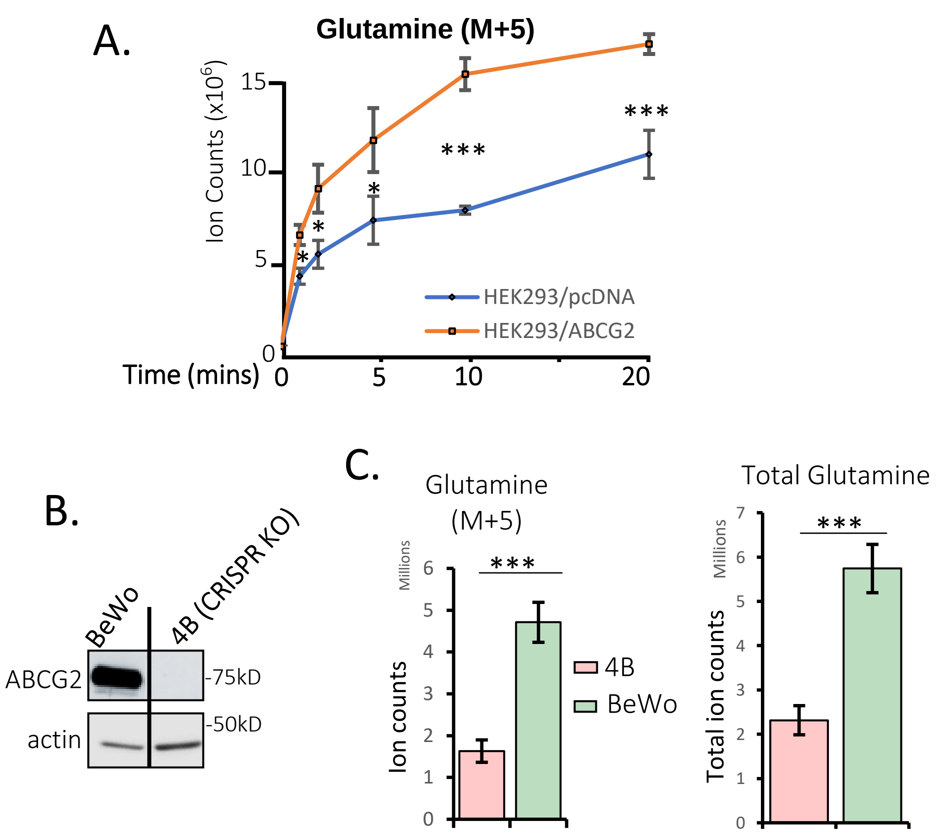

Fig S2. **ABCG2 regulates SLC1A5-mediated glutamine uptake**. (**A**) [U-$^{13}C_5$]-glutamine tracer was added to culture medium and HEK293/pcDNA and HEK293/ABCG2 cells were collected at the indicated time points post tracer addition. Glutamine uptake (glutamine (M+5)) was assessed over time by LC-MS/MS. Note the significant higher uptake of glutamine (M+5) in HEK293/ABCG2 cells compared to HEK293/pcDNA along this 20-min time course. (**B**) Western blot analysis of human placental choriocarcinoma cells that intrinsically express high levels of ABCG2 (BeWo) and BeWo cells in which ABCG2 has been knocked out using CRISPR technology (BeWo/4B). (**C**) [U-$^{13}C_5$]-glutamine tracer was added to culture medium and intracellular levels of glutamine (Glutamine (M+5)) were detected by LC-MS/MS. Note the higher glutamine uptake (left) as well as higher steady state glutamine levels (right) in BeWo cells as compared to BeWo /4B cells that lack ABCG2. Each data point represents at least 3 replicates. * indicates $p<0.05$; *** indicates $p<0.001$.

S3. ABCG2 overexpression impacts tumor cell metabolism

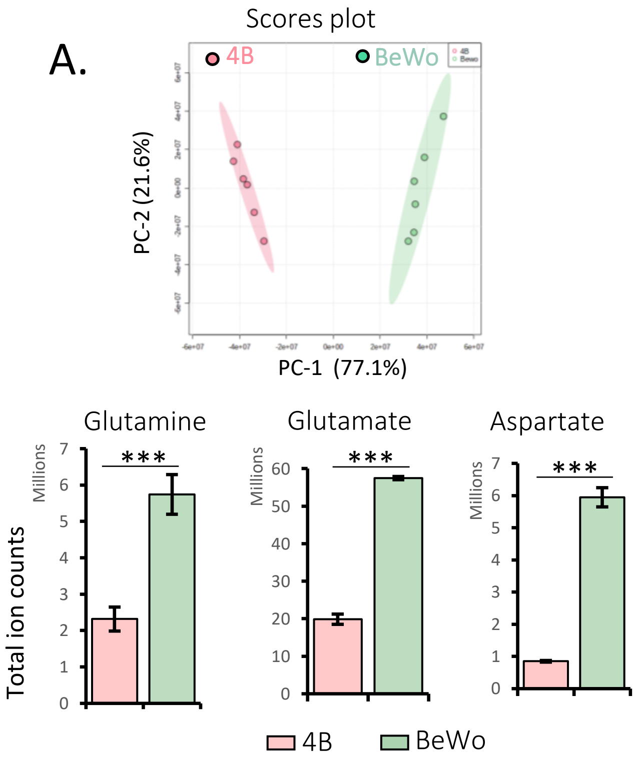

Fig S3. ABCG2 overexpression impacts tumor cell metabolism. (**A**) Principal Component Analysis revealed strikingly distinct metabolic profiles between BeWo and BeWo/4B cells, consistent with a role for ABCG2 in the phenotype. Data from six replicates of each cell type were analyzed and presented. Additional BeWo CRISPR-ABCG2 knockout clones were also analyzed with similar results (not shown). (**B**) Steady state analyses of glutamine metabolites revealed higher concentrations of glutamine, glutamate and the TCA intermediate aspartate in BeWo cells when compares to their ABCG2-KO subline BeWo/4B. *** indicates $p<0.001$.

S4. Glutamine contributes to reprogrammed metabolism in ABCG2-overexpressing cells

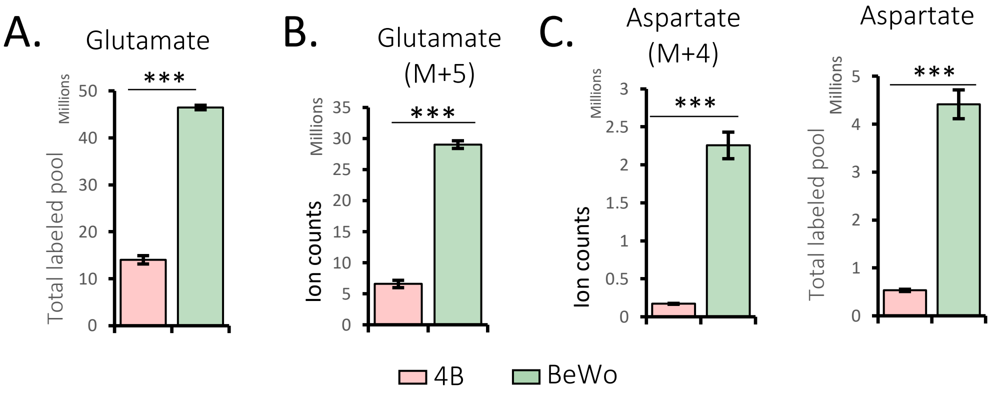

Fig S4. Glutamine contributes to reprogrammed metabolism in ABCG2-overexpressing cells. BeWo cells contain higher levels of total labeled glutamate (**A**) and the direct metabolite from glutamine (M+5), glutamate (M+5) (**B**) than the ABCG2-KO subline BeWo/4B. (**C**) BeWo cells contain higher levels of aspartate (M+4) (left), which is generated directly from glutamine (M+5) by glutaminolysis, and higher levels of total labeled aspartate (right panel), suggesting that the contribution of imported glutamine (M+5) to TCA cycle metabolites is greater in BeWo cells than in ABCG2-KO BeWo/4B cells. Data represent analyses of three replicates of each condition. *** indicates $p<0.001$.

S5. ABCG2-overexpressing cells are more resilient in the face of oxidative stress

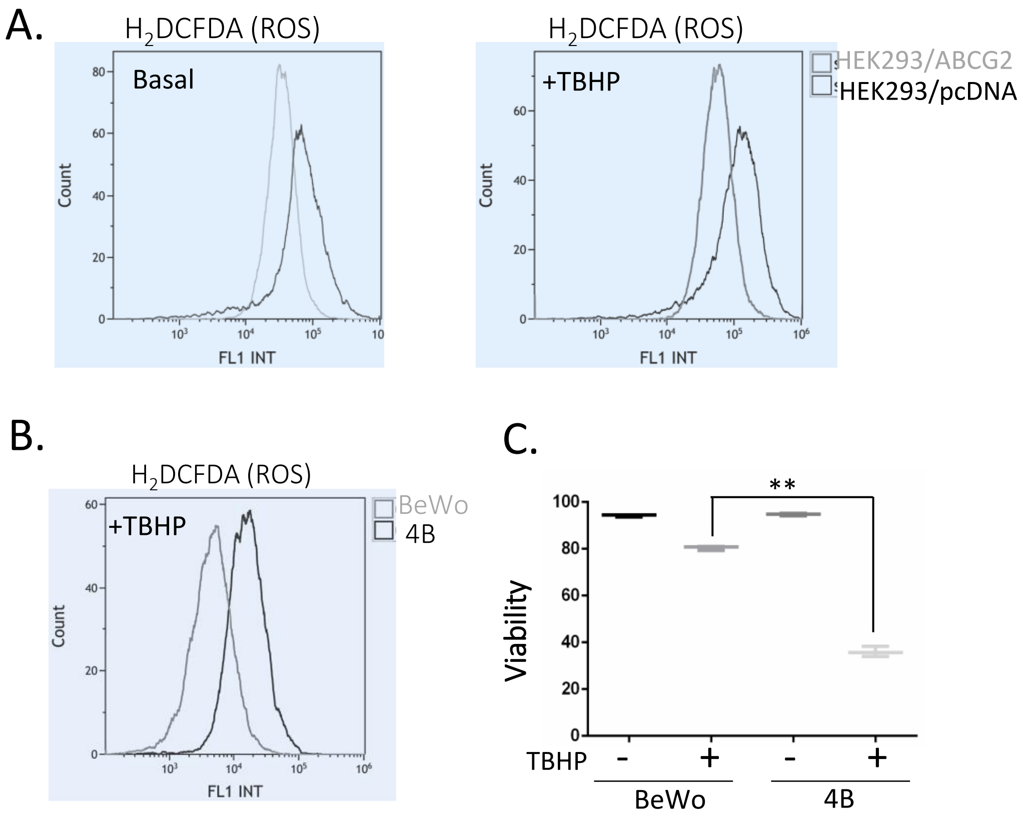

Fig S5. ABCG2-overexpressing cells are **more resilient in the face of oxidative stress.** (**A**) Lower levels of basal ROS are observed in HEK293/ABCG2 cells when compared to HEK293/pcDNA cells using a flow cytometry-based H_2DCFDA assay (left); Right -TBHP (50µM) induced higher levels of ROS in HEK293/pcDNA cells as compared to HEK293/ABCG2 cells. (**B**) ABCG2-expressing BeWo cells exhibited lower level of ROS upon TBHP treatment compared to ABCG2-KO clone BeWo/4B. (**C**) BeWo cells were treated with TBHP to induce ROS and viability was determined by trypan blue exclusion assay. ABCG2-expressing BeWo cells are more resilient to TBHP-induced ROS than 4B. Experiments were performed in triplicate. ** indicates $p < 0.01$
